# How can civil society organizations contribute to the scale-up of comprehensive sexuality education? Presentation of a scaling framework illustrated with examples from Indonesia

**DOI:** 10.1186/s12978-023-01725-6

**Published:** 2023-12-20

**Authors:** Ardan Kockelkoren, Amala Rahmah, Muhammad Rey Dwi Pangestu, Ely Sawitri, Elisabet Setya Asih Widyastuti, Ni Luh Eka Purni Astiti, Kristien Michielsen, Miranda Van Reeuwijk

**Affiliations:** 1 Rutgers, the Netherlands Centre on Sexuality, Arthur van Schendelstraat 696, 3511 MJ Utrecht, The Netherlands; 2 Rutgers Indonesia, Graha Inti Fauzi, 9th Floor, Pasar Minggu, Greater Jakarta, Indonesia; 3 PKBI Jawa Tengah, Jl. Jembawan Raya No. 8–12, Semarang, Jawa Tengah Indonesia; 4 PKBI Bali, Jl. Gatot Subroto IV No.6, Dangin Puri Kaja, Kec. Denpasar Utara, Kota Denpasar, Bali 80233 Indonesia; 5 Department of Neurosciences, Faculty of Medicine, Institute for Family and Sexuality Studies, KU Leuven, Herestraat 49, 3000 Louvain, Belgium

**Keywords:** Comprehensive sexuality education, Scaling-up, Civil Society Organizations

## Abstract

Comprehensive sexuality education (CSE) can substantially contribute to the health and well-being of young people. Yet, most CSE interventions remain limited to the small piloting or research phase and scale-up is often an afterthought at the end of a project. Because of the specificities of CSE, including it being a controversial topic in many contexts and a topic on the fringe between health, education and youth, a specific scaling approach to CSE is needed. The commentary presents a practical framework to support civil society organisations (CSOs), to address barriers to scaling up CSE in their contexts. The utilization and relevance of the framework is demonstrated in this article, by featuring examples from the scale up process of CSE in Indonesia. The framework identifies key principles for scaling up, including: taking a scaling mindset from the start, government ownership and political commitment for scale-up, and identifying the added value of CSOs. The framework starts with a self-assessment by the CSO and then follows four phases: making the case, engaging in dialogue, establishing building blocks and implementation and scale-up. Each of these phases are illustrated with examples from Indonesia.

This framework is a call to action with practical guidelines to support CSOs to take on this role, because with the right scaling strategies, the largest generation of young people ever alive can become healthy, empowered and productive adults.

## Introduction

Comprehensive sexuality education (CSE) can substantially contribute to the health and well-being of young people [1]; evaluations of CSE programs indicated clear positive effects on several aspects, including on health outcomes, equitable relationships, partner violence and safe sexual behaviors [2–4]. Furthermore, multiple studies focused on how to implement CSE effectively [5]. Yet, most CSE interventions remain limited to the small piloting or research phase and fade out after the project funding ends. The sustainability and scale-up of CSE programs are often perceived as an afterthought at the end of a project and are not commonly scheduled for from the initiation phase [6].

This has implications for young people globally, and many are left behind because of the inability to provide universal CSE coverage. Many CSE programs are implemented by civil society organizations (CSOs). If the CSO industry wants to become better at leaving no one behind and addressing the underlying structural barriers to access to CSE, this has implications for the role of CSOs and the way CSE is designed and implemented, because sustainability and scaling require different skills, approaches, and ways of collaborating than those required for successful implementation of shorter, smaller-scale community projects.

## Knowledge gap

There are several frameworks for scaling up education and health interventions, such as from Management Systems International [7], ExpandNet [8], or the Implementing Best Practices Consortium [9]. These frameworks are interesting and inspirational. However, they do not address the specific role and contribution of CSOs or the specificities of CSE, which is a controversial and contested health intervention in many countries. CSE often raises controversy, as adolescent sexuality is a taboo topic in most countries around the world [10, 11]. Furthermore, it is on the fringe of health, education, and youth, therefore oftentimes the responsibility for CSE is pushed around. CSE needs a specific scaling approach that considers these sensitivities and specificities.

In 2018, the United Nations Educational, Scientific and Cultural Organization (UNESCO) formulated ten principles that underpin successful scale-up of sexuality education, including principles as i) choose an intervention/approach that can be scaled up within existing systems, ii) clarify the aims of scaling up and the roles of different players, and ensure local/national ownership/lead role or iii) understand perceived need and fit within existing governmental systems and policies [12]. While important, these principles are often not operationalized in real-life settings [12].

Therefore, there is a need for a practical framework to support CSOs to address the barriers to scaling up CSE in their contexts by assessing the scalability of their CSE projects, designing for scale from the onset, and systematically thinking through the key elements, ingredients, and factors for success.

## Objectives and approach

Around the world, there are several examples of CSE programs that have been successfully scaled up. Based on these experiences, Rutgers, the Netherlands Centre on Sexuality, which works in 27 countries around the world, has developed a framework to support CSOs to scale their CSE programs [13]. The framework provides an overview of key steps in scale-up and issues for CSOs to consider in deciding if and how they can provide support for scale-up.

The framework is based on implementation research, program evaluations, and through firsthand experiences of direct engagement in scaling processes from a variety of countries: Benin, India, Indonesia and Zambia. For the purpose of this Commentary article, the framework will be illustrated with on the ground experiences from Indonesia.

## Key principles

A few key principles can be identified for the role CSOs can take in scaling up CSE. Firstly, taking a scaling approach from the start. Most CSO-developed CSE programmes are boutique interventions that are not scalable because they are too costly per beneficiary. Using a scaling lens from the start means collecting data/learning/adapting to develop lean programs that can reach all youth. Second, government ownership and political commitment for scale-up of CSE are critically essential. Therefore, buy-in is necessary from the policymakers, administrations, and other relevant stakeholders. CSOs often perceive the government as a black box they need to advocate against rather than build trusted relationships with. Learning how to engage, motivate, capacitate, and support civil servants is what will make the difference. Finally, CSOs should think about the added value they bring to the table in terms of technical support for scale-up. Key areas to consider are strengthening teacher training institutes and the capacities of the monitoring and examination department.

## The framework

**Fig. 1:**
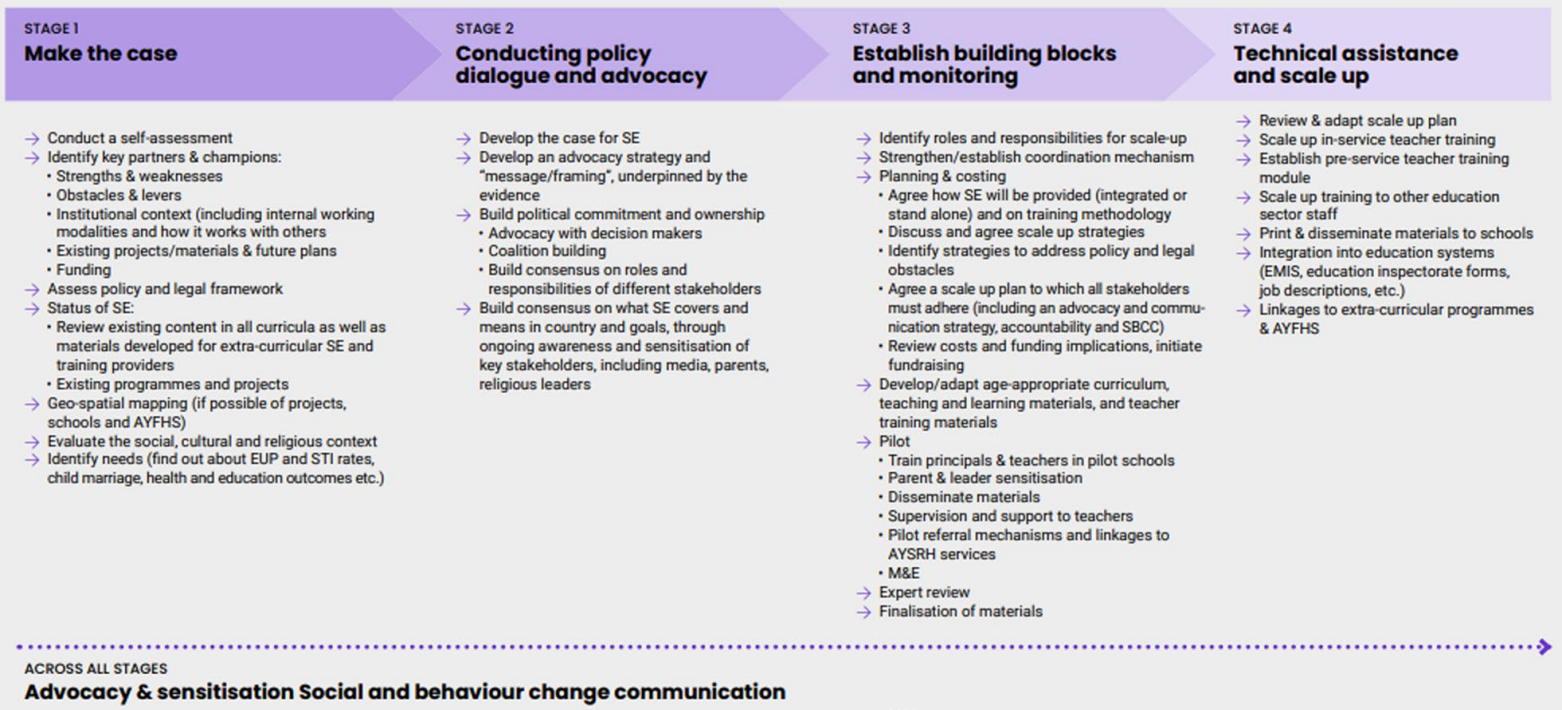
CSE scale-up framework for civil society organisations (taken from [13])

The practical framework is built around a CSE scale-up trajectory and follows four key areas where CSOs can make a valuable contribution to scaling up CSE. Here, we provide a summary of the key steps. The framework itself provides more details and practical suggestions on what CSOs can do to support CSE scale-up. A summary of the framework is provided in Fig. 1.

The framework starts with conducting a self-assessment. This self-assessment will allow a CSO to identify how it can best support CSE scale-up, as this depends on a range of factors, including the country context, the CSO’s relationship with government, its skills, expertise and capacity, its comparative advantage vis-à-vis other stakeholders, and its relationship with different stakeholders. CSOs need to consider these and other questions when deciding if they should support CSE scale-up and how they can best do this, as well as to identify whether they require additional skills and capacity.

In the next paragraphs, we will highlight key elements of the framework and present how CSOs can contribute to scaling up CSE supported by examples from Indonesia.

Stage 1: Make the Case. This stage is about understanding the context and building the case for CSE through a thorough situation analysis. This analysis can be done by a CSO and can serve as the basis for engaging in dialogue (stage 2) and developing a scale-up plan (stage 3). The situation analysis includes identifying the need for CSE, key partners, champions, and opposition, assessing the policy, legal and socio-cultural context, understanding the status of CSE and assessing the educational system and its capacity. Frequently, CSOs have a longer history of working in a particular community or geography, have an established basis of trust there, and existing relationships with the local leadership. This can be a good base to work out a cost-effective and contextually supportable local implementation model for CSE that details how the CSE intervention is embedded in policies and how it can be implemented effectively (sometimes referred to as ‘pilot model’).

In Denpasar (Bali) and Semarang (Central Java) in Indonesia, the Setara programme is implemented by local branches of Perkumpulan Keluarga Berencana Indonesia (PKBI, the Indonesian Family Planning Association), in close collaboration with Rutgers Indonesia and the city governments. The program was introduced as a way to help implement city government priorities and policies such as the ‘child-friendly schools’ policy, and ‘prevention of child sexual abuse’ [14]. Setara was positioned as a solution to address the problems that these policies want to address. Through their longstanding work in the communities in these cities and the existing relationships with their city's leadership, PKBI managed to engage in meaningful discussions about the goals, content and evidence of effectiveness with the relevant government offices in their city, resulting in a memorandum of understanding for joint coordination and implementation. Denpasar and Semarang then continued to serve as an example (‘pilot model’) for other cities and for the national government and Ministry of Education on how policies could be operationalized through decentralized systems.

Stage 2: Engaging in dialogue. This step focuses on building support for CSE. For the scale up of CSE, leadership, ownership and capacity of government decision-makers and civil servants is critical. CSOs can contribute by identifying and engaging with allies and champions from both the legislative and administrative branches of the curriculum development, teacher training and education standards units within the Ministry of Education as well as those who directly influence them. Key success factors include the perceived credibility and trustworthiness of the organizations and individuals advocating for CSE and their personal networks and connections across these cadres. The way in which CSE is framed also has a significant influence on its acceptability and the success of ensuring political ownership. Finding an entry point for CSE involves presenting CSE as a solution to contextual issues of concern and linking it to the existing priorities and KPIs of relevant institutional stakeholders. These frames are to be further supported by evidence. Evidence demonstrating the contextual need, effectiveness, financial resources, capacity and institutional changes required for CSE scale-up. Collaborations with universities and research institutions can often provide such evidence to support the rationale and provide legitimacy to the messages.

In the Setara program, Rutgers Indonesia and PKBI collaborated with the University of Gadjah Mada, center of reproductive health (UGM-CRH) to evaluate the program in Semarang and Denpasar, using the Global Early Adolescent Study (GEAS) Survey. The evaluation was technically supported by John Hopkins University, Karolinska Institutet and Rutgers [15]. Having these research partners studying the intervention helped to create legitimacy to the intervention, enhanced transparency and provided entry points to engage in dialogue about the intervention through sharing of research results with multiple stakeholders, including schools and representatives from relevant government offices (organized in ‘Local Advisory Committees'). This also brought the opportunity to bring in youth voices into the dialogue. Having UGM-CRH present about Setara's effectiveness helped to create space for a more neutral, ‘scientific’ dialogue about CSE instead of the otherwise often moral values-loaded discourse. UGM-CRH leadership was also frequently consulted by the national government as expert on Reproductive Health issues, providing an entry point to bring in CSE as a strategy to address several reproductive health priorities. Finally, Rutgers Indonesia and PKBI facilitated exchange visits to promote the Semarang and Denpasar models. They engaged media, teachers and faith leaders into these visits and built out a pool of champions for CSE. First on the city level, and later also on the national level.

Stage 3: Establish building blocks. Once political support for CSE has been established, the more practical building blocks for scaling up CSE need to be put in place. These include, amongst others, identifying all implementation partners and their roles and responsibilities (making sure CSE has an ‘institutional home’ within the education ministry), setting up coordination mechanisms, estimating the costs (what does the program cost per person, what funding is currently available and its source, as well as the funding gap and possible resource mobilization strategies), agreeing on the curriculum, delivery model (including the linkages with services and community support mechanisms) and the to be used materials, the phased roll-out plan, and clear targets. Often a Memorandum of Understanding between the CSOs, government and other collaborating institutions and organizations is a good basis for developing such a costed workplan.

In Indonesia, Setara also included a costing evaluation for its implementation in Denpasar and Semarang. The results of this evaluation were used by the implementation partners to reflect on their cost-efficiency and how they could reduce costs, and to make insightful the cost-categories. Political support for Setara had grown through the process and the city governments were now committing to further rolling out Setara to other schools in their city. Data from the costing study helped to guide division of tasks between PKBI and government departments, including who would finance what, and for lobbying budgets at the cities government level.

Stage 4: Implementation and scale-up. In this stage, the scaling plan is put into action, and CSE is integrated into existing educational systems. Teachers delivering CSE in the classroom will require training, as well as school principals, district and provincial education cadres and syllabi and examination developers. Lecturers at teacher training institutes will require training to enable a roll-out of in-service and pre-service training, and gatekeepers such as parents will need sensitization. Tools such as the annual school census, teacher lesson and school inspection forms and the Education Management Information System often need to be adapted to include CSE components and indicators [16]. Scale-up takes time and needs to be phased. Advocacy and alignment between CSOs and the government are critical to maintaining CSE in the face of opposition.

On the national level, Rutgers Indonesia, in collaboration with UNFPA, positioned itself as a technical partner to the government in achieving their key performance indicators. Political commitment was established to roll out the government-owned Reproductive Health Education curriculum, or the Setara curriculum, as a formally approved alternative, to 250 cities by 2025. A Memorandum of Understanding was established between the ministries of Health and Education, UNFPA and Rutgers Indonesia, including co-financing and division of tasks, with Rutgers Indonesia responsible for the training of teachers. Rutgers Indonesia signed memoranda of understanding with 6 district governments to jointly implement Setara with these districts’ education offices, incorporating the CSE program as a vital component of the government’s policies and strategies to reduce the prevalence of child marriage, teenage pregnancy and sexual and gender-based violence. Phase 1 (making the case and identifying partners and policies) was repeated with these districts through engaging various stakeholders and jointly thoroughly analyzing the need for CSE and developing implementation plans. Youth engagement played an important part in this process, where students provided real-life experiences of SGBV in their schools. On the national level, Rutgers Indonesia and UGM are currently facilitating workshops with government and CSE stakeholders to look at possibilities of integrating indicators for quality of implementation into the national education and management system. During COVID-19 school closures, Rutgers Indonesia developed digital solutions to continue to provide CSE to students and supporting teachers to address (digital) implementation challenges. Through the collaboration with the Ministry of Education, Rutgers Indonesia can bring in these innovations and lessons to further improve implementation at scale.

## Conclusion

Engaging in the scale-up of sexuality education requires a shift in mindset about what we perceive as our role and responsibilities as the CSO sector. A transformation is required from CSO-led CSE delivery with government support to government-led CSE delivery with CSO support. This requires acquiring different skills and applying different approaches than we are used to when successfully implementing community projects or engaging in SRHR policy advocacy. We call upon CSOs to take on a long-term scaling lens, and to invest in the development of lean and cost-effective CSE programs and in motivating, capacitating and supporting champion civil servants. This framework is a call to action with practical guidelines to support CSOs to take on this role, because with the right scaling strategies, the largest generation of young people ever alive can become healthy, empowered and productive adults.

## Data Availability

Not applicable.

## Abbreviations

CSE: Comprehensive sexuality education
CSO: Civil Society Organization
PKBI: Perkumpulan Keluarga Berencana Indonesia
SRH: Sexual and Reproductive Health
SRHR: Sexual and Reproductive Health and Rights
UGM-CRH: University of Gadjah Mada, Center of Reproductive Health
UNESCO: United Nations Educational, Scientific and Cultural Organization
